# The efficacy, safety, and pharmacokinetics/pharmacodynamics of telitacicept following efgartigimod in generalized myasthenia gravis: protocol of a randomized controlled trial

**DOI:** 10.3389/fimmu.2025.1604786

**Published:** 2025-10-22

**Authors:** Jia Li, Yuan Zhang, Yan Deng, Wenyu Li, Yiqi Wang, Feiteng Qi, Qiaoyi Zhang, Bingbing Wan, Xiang Li, Yiyun Weng, Zheyu Fang, Yu Zhang, Xi Qu, Shengli Pan, Shiyin Yang, Xu Zhang

**Affiliations:** ^1^ Department of Neurology, The First Affiliated Hospital of Wenzhou Medical University, Wenzhou, Zhejiang, China; ^2^ Department of Neurology, Second Affiliated Hospital, School of Medicine, Zhejiang University, Hangzhou, Zhejiang, China; ^3^ Department of Neurology, Sir Run Run Shaw Hospital, School of Medicine, Zhejiang University, Hangzhou, Zhejiang, China; ^4^ Department of Neurology, Zhejiang Provincial People’s Hospital, Hangzhou, Zhejiang, China; ^5^ Department of Neurology, Ningbo Medical Center Lihuili Hospital, Ningbo, Zhejiang, China

**Keywords:** generalized myasthenia gravis, telitacicept, efgartigimod, BLyS/APRIL inhibitor, PK/PD

## Abstract

**Introduction:**

Several biologic agents have emerged as novel therapeutic options for patients with generalized myasthenia gravis (gMG); however, no clinical studies have yet explored the efficacy and safety of sequential biologic therapy in gMG.

**Methods and analysis:**

This multicenter, open-label, randomized controlled, exploratory clinical trial plans to enroll 60 patients with acetylcholine receptor antibody-positive gMG, randomized in a 1:1:1 ratio to receive one of the following treatment regimens: (1) E + 1w+T: efgartigimod 10 mg/kg weekly for 4 weeks, followed by telitacicept 240 mg weekly starting 1 week after the last efgartigimod dose, continued for 25 weeks; (2) E + 2w+T: efgartigimod as above, followed by telitacicept 240 mg weekly starting 2 weeks after the last efgartigimod dose, continued for 24 weeks; or (3) T only: telitacicept monotherapy for 30 weeks. The primary endpoint is the change in the Quantitative Myasthenia Gravis (QMG) score from baseline to week 30. Secondary endpoints include changes in the Myasthenia Gravis Activities of Daily Living (MG-ADL) score from baseline, proportion of patients achieving minimal manifestation status (MMS), changes in dosages of corticosteroid and other immunosuppressant, rates of MG relapse/acute exacerbation and MG crisis, and safety outcomes. The pharmacokinetics/pharmacodynamics (PK/PD) of telitacicept will also be assessed. Recruitment is currently ongoing, but no participants have been enrolled as of yet.

**Ethics and dissemination:**

The study has been approved by the Ethics Committee in Clinical Research of the First Affiliated Hospital of Wenzhou Medical University. Results of the study will be disseminated to the relevant scientific, clinical and patient communities on trial closure.

Trial registration number: The study was registered at ClinicalTrials.gov (NCT06827587).

## 1 Introduction

Myasthenia gravis (MG) is an autoimmune neuromuscular disorder mediated by autoantibodies, characterized primarily by localized or generalized muscle weakness and fatigability (1, 2). A recent systematic review estimated the global prevalence of MG to be approximately 173.3 per million, with an annual incidence of 15.7 per million (3). In China, the age- and sex-adjusted annual incidence of MG is approximately 0.68 per 100,000, with an in-hospital mortality rate of 1.469% (4). Approximately 85% of patients develop generalized myasthenia gravis (gMG), which predominantly affects the proximal muscles of the limbs and trunk (2, 4). Myasthenic crisis occurs in 15-20% of patients with gMG, often leading to respiratory failure and bulbar palsy, and requiring intensive care (1, 5).

The pathogenesis of MG is primarily mediated by immunoglobulin G (IgG) autoantibodies targeting postsynaptic membrane receptors, impairing neuromuscular transmission (5). Acetylcholine receptor antibodies (AChR-Ab) are the most prevalent, detected in about 80% of MG cases (1, 5). A smaller proportion of patients have antibodies against muscle-specific tyrosine kinase (MuSK) or low-density lipoprotein receptor-related protein 4 (LRP4). Standard treatment includes cholinesterase inhibitors, corticosteroids, and conventional immunosuppressants, along with intravenous immunoglobulin (IVIg) or plasma exchange (PLEX) for acute exacerbations (2, 6, 7). However, current therapies are limited by delayed onset of action, broad adverse effect profiles, unstable symptom control during corticosteroid tapering, and high relapse rates (8). Many gMG patients fail to achieve minimal manifestation status (MMS) promptly and persistently or endure chronic treatment-related morbidity. Thus, there remains a critical unmet need for safe, effective, and durable therapies that provide early disease control and reduce long-term immunosuppressant exposure.

In recent years, novel biologic agents have significantly expanded the treatment landscape for gMG. These include complement inhibitors (e.g., eculizumab), neonatal Fc receptor (FcRn) antagonists (e.g., efgartigimod), and B-cell-targeted therapies (e.g., telitacicept) (5). Eculizumab was approved in China in 2023 for anti-AChR-Ab-positive refractory gMG, though symptom worsening upon discontinuation has been reported (9). Efgartigimod, approved in China in September 2023, rapidly alleviates symptoms by promoting IgG degradation through FcRn inhibition (10, 11). Both intravenous and subcutaneous formulations have now been approved by the U.S. Food and Drug Administration, the European Medicines Agency, and more recently by Chinese regulatory authorities for the treatment of gMG. Yet its clinical benefit may be short-lived, with symptom rebound linked to anti-AChR antibody overshoot and a relatively short half-life (4.89 days) (12–14). Real-world studies indicate that scores such as Quantitative Myasthenia Gravis (QMG) and Myasthenia Gravis Activities of Daily Living (MG-ADL) begin to rebound within 2–3 weeks of completing a 4-week cycle (10, 14). Because subsequent treatment cycles cannot begin until 7 weeks post-initiation, the need for a durable maintenance strategy after efgartigimod-induced remission is pressing. Telitacicept, a recombinant fusion protein targeting BLyS and APRIL, inhibits B-cell maturation and plasma cell differentiation, thereby reducing autoantibody production (15, 16). It is approved in China for systemic lupus erythematosus and has shown promising efficacy and tolerability in gMG. A multicenter phase 2 trial reported sustained QMG and MG-ADL improvements over 24 weeks (15). A 2024 retrospective study showed that 90.1% of patients with refractory gMG experienced sustained clinical benefit and corticosteroid dose reduction after 6 months of telitacicept therapy (17). Its subcutaneous route also facilitates long-term outpatient administration.

Mechanistically, efgartigimod and telitacicept act on complementary immunologic pathways. Efgartigimod provides rapid, downstream clearance of pathogenic IgG (10, 18), while telitacicept offers prolonged, upstream suppression of autoantibody production (16). Therefore, sequential use may enable both rapid symptom control and long-term disease stabilization. Furthermore, this biologic induction-maintenance model may facilitate early corticosteroid tapering and reduce reliance on long-term immunosuppression. In our previous case series, seven patients who responded poorly to conventional therapies demonstrated significant improvement in QMG and MG-ADL scores following treatment with telitacicept and efgartigimod, with no reported adverse events (19). These preliminary findings support the safety and feasibility of sequential biologic therapy. Nevertheless, one unresolved question is the optimal timing of telitacicept initiation after efgartigimod. As a human IgG Fc fusion protein, telitacicept could be prematurely catabolized by residual efgartigimod, potentially reducing its bioavailability. If administered too late, however, symptom rebound may occur before telitacicept becomes effective. Based on prior pharmacokinetic modeling and clinical experience, we decided to evaluate both 1-week and 2-week intervals between therapies.

To address this clinical gap, we propose a multicenter, open-label, randomized controlled trial comparing two sequential efgartigimod-telitacicept regimens versus telitacicept monotherapy in patients with AChR-Ab-positive gMG. This study will assess efficacy, safety, and pharmacokinetics/pharmacodynamics (PK/PD) parameters, with the aim of defining an optimal sequencing strategy. This approach could represent a novel treatment paradigm for patients with refractory gMG, offering a biologics-based induction-maintenance model to improve early disease control and reduce corticosteroid dependency.

## 2 Methods and analysis

### 2.1 Study design

This study is a multicenter, open-label, randomized controlled clinical trial which will be conducted at five centers, including the First Affiliated Hospital of Wenzhou Medical University. The study will adhere to Good Clinical Practice standards and the principles of the Declaration of Helsinki. The protocol has been approved by the Ethics Committee in Clinical Research of the First Affiliated Hospital of Wenzhou Medical University (KY2024-298) and has been registered at ClinicalTrials.gov (NCT06827587).

### 2.2 Study subjects

The study will enroll patients aged 18–80 years with AChR-Ab-positive gMG, classified as Myasthenia Gravis Foundation of America (MGFA) Class II-IV [15]. Full eligibility criteria are provided in **Table 1**.

**Table 1:** Inclusion and exclusion criteria.

Inclusion criteria
1. Patients must voluntarily sign the informed consent form.
2. Age between 18 and 80 years, inclusive, with no sex restrictions.
3. Diagnosis of MG according to the 2020 Chinese Guidelines for the Diagnosis and Treatment of MG, with serologically confirmed AChR-Ab positivity.
4. Classified as Myasthenia Gravis Foundation of America (MGFA) clinical class II–IV.
5. Patients with fluctuating MG symptoms before enrollment, defined as an MG-ADL score ≥6 or a Quantitative Myasthenia Gravis (QMG) score ≥8, persisting for more than 24 hours.
Exclusion criteria
1. Presence of other active autoimmune diseases, such as systemic lupus erythematosus, rheumatoid arthritis, or Sjögren’s syndrome.
2. Active infections, including herpes zoster, HIV, active tuberculosis, or active hepatitis.
3. Thymoma with a history of surgery within the past six months.
4. History of malignancies other than thymoma.
5. Severe hepatic or renal impairment, defined as ALT or AST >3×ULN, or an estimated glomerular filtration rate (GFR) <30 mL/min/1.73 m².
6. Serum IgG level ≤400 mg/dL.
7. Prior use of biologic agents within five times their elimination half-life, including: Telitacicept within two months prior to enrollment. Efgartigimod within one month prior to enrollment. Rituximab or other targeted biologic therapies within six months prior to enrollment.
8. Use of intravenous immunoglobulin (IVIG) or plasma exchange within two months before enrollment.
9. Receipt of any live vaccine within three months prior to enrollment or planned vaccination during the study period.
10. Pregnant or lactating women and those planning to conceive during the study period.
11. Known hypersensitivity to human-derived biologic products.
12. Participation in any clinical trial within 28 days before enrollment or within five times the elimination half-life of the investigational drug.
13. Other conditions deemed inappropriate for study participation by the investigator (e.g., severe psychiatric disorders).

### 2.3 Randomization and intervention

**Figure 1:**
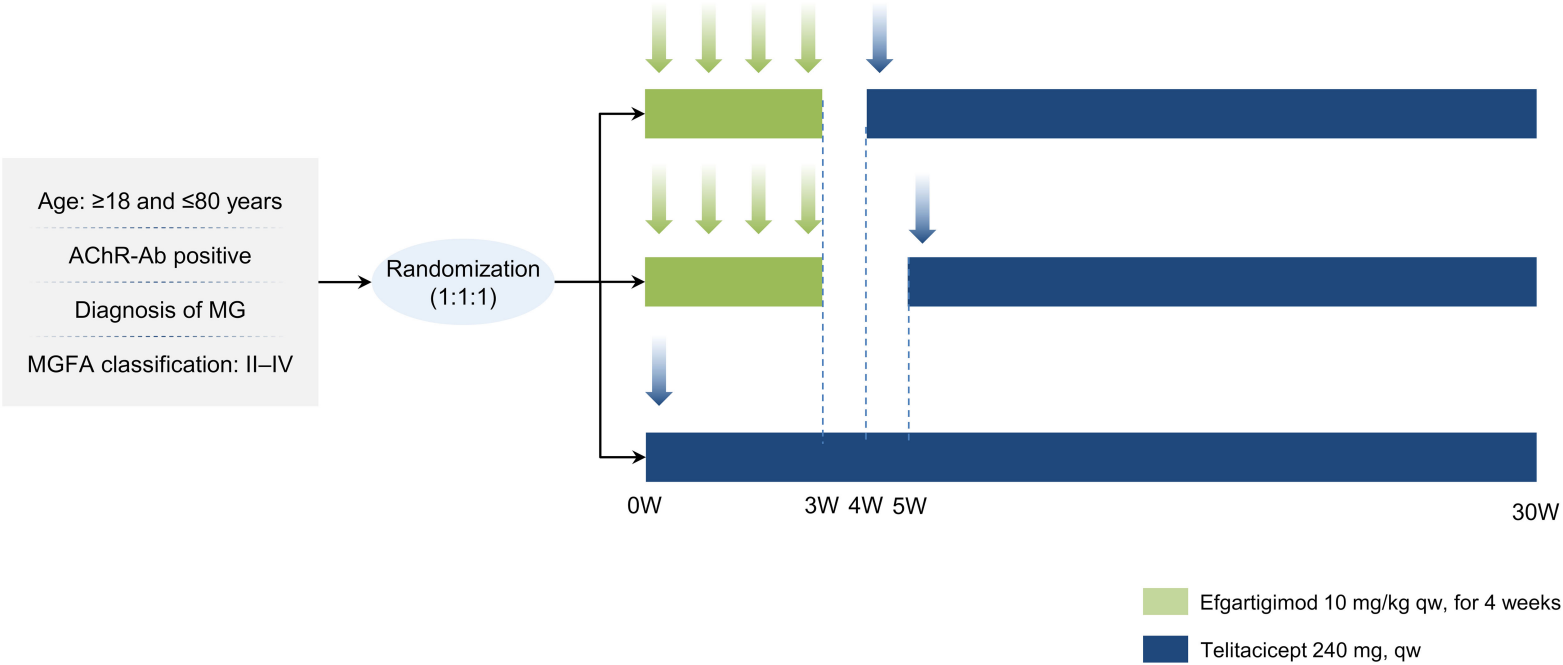
Study flowchart. MG, myasthenia gravis.

Randomization will be conducted using a computer-generated random sequence, with all assignments managed through a central randomization system to ensure allocation concealment. Eligible participants will be randomly assigned in a 1:1:1 ratio to one of three groups, with 20 patients per group: E + 1w+T group: Efgartigimod followed by telitacicept with a 1-week interval; E + 2w+T group: Efgartigimod followed by telitacicept with a 2-week interval; T only group: Telitacicept monotherapy. All participants will receive biologic therapy in addition to standard-of-care treatments, which include acetylcholinesterase inhibitors, corticosteroids, and non-steroidal immunosuppressants such as tacrolimus. The study flowchart is shown in **Figure 1**.

E+1w+T group: Participants will receive efgartigimod induction therapy at 10 mg/kg, administered via intravenous infusion over 1 hour, once weekly for a total of 4 consecutive doses (Weeks 0–3). One week after completing induction therapy, telitacicept maintenance therapy will be initiated at a dose of 240 mg, administered subcutaneously once weekly from Week 4 for a total of 25 weeks. Participants will be followed until Week 30.

E+2w+T group: Participants will receive efgartigimod induction therapy at 10 mg/kg via intravenous infusion, once weekly for a total of 4 consecutive doses (Weeks 0–3), as in the E + 1w+T group. However, telitacicept maintenance therapy (240 mg, subcutaneously once weekly) will be initiated two weeks after completing induction therapy, starting from Week 5, and will continue for 24 weeks. Participants will be followed until Week 30.

T only group: Participants will not receive efgartigimod induction therapy. Instead, they will receive telitacicept 240 mg subcutaneously once weekly from Week 0 for a total of 30 weeks. Participants will be followed until Week 30.

From Week 8 to Week 30, if the patient reaches the Minimal Symptom Expression (MSE) criterion (MG-ADL ≤ 1) or experiences significant improvement in the Post-Intervention Status (PIS), or if the investigator deems dose reduction necessary, a reduction in the standard treatment is allowed. The recommended sequence for dose reduction is to first reduce or discontinue pyridostigmine bromide, followed by a reduction or discontinuation of non-steroidal immunosuppressive agents (e.g., tacrolimus, azathioprine, mycophenolate mofetil), and finally a reduction or discontinuation of corticosteroids. However, this order may be adapted at the discretion of the investigator based on the patient’s clinical condition, medication tolerance, and treatment response. To ensure stable disease control, a 2–4 week interval should be observed between the reduction or discontinuation of each class of medication. If disease exacerbation occurs during the study (MG-ADL increase≥2 points), but the patient does not reach a crisis state, an increase in the standard treatment or the use of a rescue regimen is permitted.

### 2.4 Rescue treatment for MG exacerbation or crisis

In the event of a myasthenic crisis (V-type) during the study, investigational treatment will be immediately discontinued. The investigator will initiate appropriate rescue therapies, which may include, but are not limited to: 1) High-dose corticosteroids, such as methylprednisolone 1000 mg/day intravenously for 3 consecutive days, followed by a tapering regimen. Each infusion should be administered over 3–4 hours to reduce cardiac adverse effects; 2) IVIG at 400 mg/kg/day for 5 consecutive days; 3) PLEX, typically performed every other day during the first week (3 sessions), followed by weekly sessions depending on clinical response, for a total of 5–7 treatments. Each session may include replacement with approximately 1500 mL of fresh-frozen plasma and 500 mL of plasma substitute; 4) Supportive measures, including stabilization of vital signs, respiratory support, and symptomatic treatment for comorbid conditions as necessary, provided they do not interfere with efficacy evaluation.

For patients who experience moderate exacerbation (e.g., MG-ADL increase ≥2 points without meeting crisis criteria), the investigator may escalate standard therapy or initiate rescue treatment per clinical judgment. All adverse events and therapeutic interventions will be documented per protocol.

### 2.5 Criteria for discontinuing

Patients have the right to withdraw from the study at any time for any reason. The investigator has the authority to terminate a patient’s participation under the following circumstances: pregnancy; receipt of rescue treatment; occurrence of adverse events that make the patient unsuitable for continued participation; significant laboratory abnormalities; serious violations of the study protocol; loss to follow-up; study termination due to management or other reasons; the investigator’s assessment that the patient is not benefiting from the study; or if continued participation poses unacceptable risks to the patient.

### 2.6 Endpoints

The primary endpoint is the change from baseline in the QMG score at Week 30 after randomization. Secondary endpoints include the assessment of the following variables at Weeks 4, 8, 12, 18, 24, and 30 after randomization: change from baseline in the MG-ADL score; the proportion of patients achieving minimal manifestation status (MMS) (defined as the absence of any functional limitations due to myasthenia, with certain weakness detected by a trained neurologist); the proportion of patients with a reduction of ≥2 points in the MG-ADL score from baseline; the proportion of patients with a reduction of ≥3 points in the QMG score from baseline; changes in the dose of corticosteroids and other immunosuppressive agents at Weeks 24 and 30; the proportion of patients who discontinue corticosteroids and other immunosuppressive agents at Weeks 24 and 30; the proportion of patients on prednisone (or equivalent corticosteroids) ≤5 mg/day at Weeks 24 and 30; the incidence of MG relapse/acute exacerbation and MG crisis at Week 30; and the incidence of AEs and SAEs.

The study will also analyze the PK/PD characteristics of telitacicept in different sequential treatment groups. Blood samples will be collected to measure PK-related indicators before the first administration of telitacicept (30 minutes prior), and at 6, 24, 48, and 72 hours, as well as at Week 1, 4, 8, 12, and 24 (before each dose). The PK parameters to be measured include blood drug concentration, clearance rate, volume of distribution, interindividual variability, and other relevant indicators. Immune globulins will be tested during the screening period, during the first cycle of efgartigimod treatment, prior to the first dose of telitacicept, and at Weeks 1, 4, 8, 12, and every 4 weeks thereafter, until the end of the study. During the screening period, prior to the first dose of telitacicept, and at Weeks 4, 12, and 24 of telitacicept treatment, BLyS/APRIL, B-cell flow cytometry, T-cell flow cytometry, and cytokines such as IL-6 will be assessed, all before dosing.

The evaluation of adverse events will adhere to the NCI-CTCAE V5.0.

### 2.7 Sample size calculation

Based on previous studies (15), the change in QMG score from baseline at 24 weeks in the monotherapy group was -9.6 (± 4.3). It is assumed that the efficacy of the two sequential treatment regimens in this study will be similar, with a predicted additional 4-point reduction in the QMG score at 30 weeks for the sequential treatment group compared to the monotherapy group (10, 19), with SD = 4.3. Using a two-sided test, α = 0.025, and power = 80%, ANCOVA will be used for pairwise comparisons, assuming R2 = 0.3. The required sample size per group is 17 participants. Considering a 15% dropout rate, 20 participants per group are planned, for a total of 60 participants.

### 2.8 Data collection and management

All data will be recorded in an electronic case report form (eCRF) by investigators or clinical research coordinators. Completed CRFs will be submitted to the respective study centers for archiving. All study documents will be considered confidential. The research unit is responsible for maintaining all study materials, including confirmation of all participants (to effectively verify different records, such as research case files), original signed informed consent forms, and detailed records of drug distribution, until 5 years after the completion of the trial. In addition to MG-specific data, participants’ comorbidities and concomitant medications for non-MG-related conditions will be recorded.

### 2.9 Statistical method

This study will analyze data based on the intention-to-treat (ITT) principle. Efficacy will be evaluated based on the full analysis set (FAS), and safety will be evaluated based on the safety analysis set (SS). FAS is defined as all participants who were randomly assigned, received at least one dose of the study drug, and have efficacy evaluations. The safety analysis set is defined as all participants who were randomly assigned, received at least one dose of the study drug, and have safety assessments, summarized by the actual treatment received.

The primary endpoint, the change in QMG score from baseline at 30 weeks, will be compared using ANCOVA for E + 1w+T vs. T only and E + 2w+T vs. T only, adjusting for baseline QMG score. Missing values for the primary endpoint will be handled using the last observation carried forward (LOCF) method. Secondary endpoints will be analyzed according to general statistical principles. Two-sided tests will be used with a significance level of 5%.

## 3 Discussion

MG is an antibody-mediated autoimmune disease, with B cells playing a key role. B cell-depleting agents have the potential to revolutionize the MG treatment landscape, though these agents are still under investigation (20, 21). Previous studies suggest that intensified immunotherapy increases the MMS achievement rate in MG patients, allowing for steroid dose reduction. However, this is limited by the use of high-dose steroids, IVIG, and plasma exchange, and there is a lack of prospective studies. Efgartigimod, similar to plasma exchange, targets downstream pathogenic pathways in MG, rapidly clearing pathogenic IgG to induce disease remission (22). Telitacicept inhibits upstream B cell differentiation and antibody production. Clinical trials indicate that telitacicept is effective and safe in treating gMG (15). There is substantial evidence supporting the efficacy and safety of efgartigimod in treating gMG, but maintaining efficacy remains a key clinical concern. Recent case reports suggest that combining targeted B cell therapies may address this issue (19). Currently, there are no prospective clinical studies on biologic sequential treatment for MG. This study will be the first to explore the efficacy and safety of telitacicept sequentially following efgartigimod treatment. Additionally, since efgartigimod may promote the metabolism of monoclonal antibodies, the optimal interval between sequential treatment with efgartigimod and telitacicept remains uncertain. This study will also investigate the impact of efgartigimod sequential treatment on telitacicept’s PK/PD.

Efgartigimod, by blocking the FcRn, promotes rapid degradation of circulating pathogenic IgG antibodies, providing fast symptom relief. However, its effect is pharmacodynamically transient, with a short half-life of approximately 3–5 days, and current clinical protocols do not support indefinite use (12, 13). Consequently, disease exacerbation following efgartigimod discontinuation has been observed. In contrast, telitacicept is a dual BLyS/APRIL inhibitor that acts upstream by inhibiting B-cell maturation and plasma cell differentiation, leading to reduced autoantibody production (15–17). It has a favorable pharmacologic profile for chronic administration and has not been associated with rebound phenomena after discontinuation. A recent real-world case series reported sustained clinical stability in patients with gMG for at least eight weeks following telitacicept withdrawal (23). The current study design therefore aims to harness the rapid but short-lived benefits of efgartigimod induction, followed by long-acting telitacicept to provide durable disease control and minimize relapse risk during and after treatment tapering or discontinuation.

Although both efgartigimod and telitacicept act on the humoral immune axis, their PD profiles differ significantly. Efgartigimod promotes rapid but reversible IgG clearance via FcRn inhibition, with serum IgG levels typically rebounding within weeks after discontinuation (10). Telitacicept, a BLyS/APRIL dual inhibitor, reduces IgG production gradually by impairing B-cell differentiation, with clinical and immunologic onset typically occurring around 4 weeks into therapy (15, 24). To mitigate risks of excessive IgG reduction, the trial includes a conservative eligibility criterion of serum IgG >400 mg/dL, consistent with safety guidelines in telitacicept’s prescribing information. Immunoglobulin levels will be regularly monitored, and dose interruption criteria are pre-specified to ensure patient safety. Moreover, retrospective data from our research group on sequential therapy use in refractory gMG did not reveal any safety signals of IgG over-suppression or increased infection risk (19, 25). Together, these measures provide a robust framework to ensure participant safety while enabling investigation of this promising sequential approach.

In the efgartigimod phase 3 clinical trial and the extended observation ADAPT+ study, patients showed a rebound in disease scores, such as MG-ADL and QMG, 2 weeks after completing one treatment cycle, with scores returning to baseline levels 4–5 weeks after discontinuation (10). A multicenter, randomized, open-label phase 2 clinical trial of telitacicept in patients with gMG indicated that the efficacy of telitacicept is significant after 4 weeks of administration (15). Therefore, in this study, the initiation of telitacicept in one sequential treatment group will be set to 1 week after efgartigimod discontinuation to avoid symptom rebound due to prolonged treatment gaps. Considering the theoretical degradation effect of efgartigimod on the IgG Fc fusion protein in telitacicept, the other sequential treatment group will begin telitacicept treatment 2 weeks after efgartigimod administration, following 3 half-lives of efgartigimod (13), to avoid the influence of residual drug concentrations on subsequent treatment while stabilizing patients’ symptoms as far as possible. The study will compare two sequential treatment groups with different time intervals, conducting a multidimensional assessment of efficacy, safety, and PK/PD characteristics to explore the optimal sequential treatment strategy.

This study is the first clinical trial to explore sequential treatment strategies for gMG. One of the strengths of this study is its randomized, parallel-controlled design. Another advantage is the inclusion of PK/PD assessments, in addition to efficacy and safety data. The design of the PD indicators is based on the pharmacological mechanisms of efgartigimod and telitacicept, including immunoglobulin, B cell count, and/or BLyS+APRIL levels. Efgartigimod promotes IgG clearance (18), while telitacicept, a dual inhibitor of BLyS/APRIL, inhibits B cell maturation and suppresses the secretion of autoantibodies by blocking BLyS and APRIL (16). Monitoring these relevant PD indicators allows the study to analyze the rationale behind the sequential treatment strategy from a pharmacodynamic perspective.

The QMG score was selected as the primary endpoint in this study due to its objectivity, granularity, and sensitivity in assessing changes in muscle strength across a broad range of functional domains. Compared to MG-ADL, the QMG provides a more detailed and examiner-rated evaluation of disease severity, which is particularly useful in early-phase trials where subtle treatment effects may be more readily detected. Furthermore, the QMG has been used as the primary efficacy endpoint in previous clinical studies of biologic therapies in gMG (26). While MG-ADL is a valuable patient-reported outcome, it has been included as a secondary endpoint to ensure a comprehensive understanding of both clinical and patient-experienced treatment effects.

Corticosteroid tapering in patients with gMG requires careful clinical judgment to avoid symptom rebound or disease instability. In the current trial, prednisone tapering is guided by strict clinical criteria, including the achievement of MMS or other markers of sustained symptom control. The protocol allows for individualized tapering and mandates a minimum 2–4 week interval between adjustments to ensure patient safety. Prior studies involving telitacicept have demonstrated that corticosteroid dose reduction is feasible in the context of biologic therapy (19, 25). Our study builds on this evidence and incorporates safety mechanisms such as continued background immunosuppressive therapy and predefined criteria for rescue intervention. As steroid tapering is an exploratory secondary endpoint, its outcomes will be carefully analyzed to inform future clinical decision-making.

This study has several limitations. First, the complex treatment regimen is open-label and does not employ blinding, as the three groups receive distinct treatment regimens that make blinding unfeasible, which may introduce measurement bias despite the use of objective endpoints such as the QMG score. Second, the relatively small sample size limits statistical power and generalizability; larger confirmatory trials will be needed to validate the findings. Third, while the combination of efgartigimod and telitacicept raises a theoretical concern regarding excessive IgG reduction, current PK and PD data suggest that these agents, due to their distinct mechanisms and temporal profiles, are unlikely to produce synergistic over-suppression. Nevertheless, IgG levels will be monitored closely to ensure patient safety. Additionally, the protocol includes a relatively ambitious immunosuppressant tapering strategy, allowing for reduction of both corticosteroids and non-steroidal immunosuppressants during the 30-week treatment period. Flexibility has been built into the protocol, allowing for individualized tapering decisions and prompt initiation of rescue therapy in cases of symptom worsening or myasthenic crisis. Finally, while the exploratory nature of this study limits its capacity to draw definitive conclusions, it may serve as a proof of concept for sequential biologic therapy in gMG.

This study provides clinical research evidence for subsequent sequential treatment strategies in gMG patients treated with biologics, aiming to further improve patient prognosis.

### 3.1 Trial status

The trial has been registered in ClinicalTrials.gov (Registration number: NCT06827587; registration date: 2025-02-14; https://clinicaltrials.gov). Recruitment is currently ongoing, but no participants have been enrolled as of yet. The protocol version number is 1.0.
